# Adverse event risks and prognostic factors associated with endoscopic laryngopharyngeal surgery for superficial head and neck cancer after chemoradiotherapy

**DOI:** 10.1007/s10147-026-03186-w

**Published:** 2026-09-19

**Authors:** Sakiko Naito, Hayato Yamaguchi, Masakatsu Fukuzawa, Isaku Okamoto, Kiyoaki Tsukahara, Takao Itoi

**Affiliations:** 1 Department of Gastroenterology and Hepatology, Tokyo Medical University, 6-7-1, Nishinjuku, Shinjuku-ku, Tokyo, 160-0023 Japan; 2 Department of Otorhinolaryngology, Head and Neck Surgery, Tokyo Medical University, 6-7-1, Nishinjuku, Shinjuku-ku, 160-0023 Tokyo, Japan

**Keywords:** ELPS, Head and neck cancer, Salvage

## Abstract

**Background:**

Endoscopic laryngopharyngeal surgery (ELPS) is an organ-preserving treatment for superficial head and neck squamous cell carcinoma (sHNSCC). While ELPS shows favorable outcomes in primary and metachronous lesions, its role as salvage treatment following chemoradiotherapy (CRT) remains unclear. We evaluated the clinical outcomes of salvage ELPS and factors associated with adverse events (AEs) and prognosis.

**Methods:**

Among 136 patients undergoing ELPS between 2014 and 2024, we retrospectively analyzed 17 diagnosed with sHNSCC following CRT. Patients were categorized into salvage and metachronous groups, and clinical characteristics, treatment outcomes, AEs, and survival were compared.

**Results:**

The en bloc resection rate was 100%. AEs occurred in 47.1% of patients and were more frequent in the salvage group than in the metachronous group (70.0% vs. 14.3%, *p*=0.024). Body mass index (BMI) <18.5 kg/m^2^ was significantly associated with AEs (*p*=0.043). 4-year overall survival (OS) did not differ significantly between groups (83.3% vs. 71.4%), while 12-month relapse-free survival tended to be lower in the salvage group (68.6% vs. 85.7%). BMI was also associated with OS: patients with BMI ≥18.5 kg/m^2^ had a 4-year OS of 81.8%, compared with 50% for patients with BMI <18.5 kg/m^2^ (*p*=0.036).

**Conclusion:**

Salvage ELPS for sHNSCC was associated with a higher AE risk but showed long-term survival outcomes comparable to metachronous ELPS. Nutritional status, particularly low BMI, is a key determinant of safety and prognosis. These findings support the feasibility of ELPS as an organ-preserving strategy and emphasize the importance of careful patient selection and nutritional assessment in salvage settings.

## Introduction

Head and neck squamous cell carcinoma (HNSCC) is a major global health burden, accounting for >900,000 new cases and >400,000 deaths annually [1]. Although multimodal treatments, including surgery and chemoradiotherapy (CRT), have improved survival outcomes, treatment-related functional impairments, particularly dysphagia and voice dysfunction, remain critical determinants of long-term patient quality of life. CRT has been established as the standard of care for organ preservation [2, 3]; however, recurrence occurs in 25–48% of patients, making salvage therapy an important component of disease management [4, 5].

The high incidence of acute and long-term toxicities following CRT [6] contributes to increased postoperative complications and mortality, which may limit the feasibility of salvage surgery [7–9]. Etiological factors also vary by region: while human papillomavirus-related disease predominates in Western countries [10], HNSCC in Japan is more commonly associated with environmental exposures such as tobacco use and genetic polymorphisms in alcohol-metabolizing enzymes [11, 12]. Acetaldehyde exposure promotes field cancerization, increasing the risk of both HNSCC and esophageal squamous cell carcinoma (ESCC) [11–13], and leading to frequent synchronous and metachronous carcinogens.

Given these challenges, strategies that preserve organ function without compromising oncological control are of substantial clinical importance. Endoscopic laryngopharyngeal surgery (ELPS), pioneered in Japan, has emerged as an effective organ-preserving treatment for superficial HNSCC (sHNSCC), offering favorable local control while preserving laryngeal function [14, 15]. However, its application in the post-CRT setting presents unique challenges. Radiation-induced tissue fibrosis, mucosal fragility, edema, and xerostomia may increase procedural complexity and the risk of postoperative complications, potentially resulting in nutritional deterioration and poorer clinical outcomes [16].

Despite the growing need for salvage interventions in patients with HNSCC after CRT, evidence regarding the safety, efficacy, and prognostic determinants of ELPS after CRT remains scarce. Addressing this gap is essential for optimizing treatment selection and risk stratification. Therefore, in this study, we evaluated the clinical outcomes of ELPS for sHNSCC following CRT and investigated clinical factors associated with adverse events (AEs) and prognosis to inform treatment strategies for post-CRT salvage surgery, considering preoperative risk factors.

## Patients and methods

### Patients and study design

This retrospective study included 17 patients diagnosed with sHNSCC following CRT among 136 patients who underwent ELPS at Tokyo Medical University Hospital (Tokyo, Japan) between 2014 and 2024. Of these, 10 and seven underwent salvage and metachronous ELPS, respectively. Salvage ELPS was defined as ELPS performed for recurrent or persistent disease arising within a previously irradiated field after radiotherapy or CRT. Metachronous ELPS was defined as ELPS performed for a newly developed lesion occurring at a temporally distinct time point after curative CRT. Operationally, salvage ELPS denoted residual or recurrent lesions detected within 1 year of completing (chemo)radiotherapy, whereas metachronous ELPS denoted newly developed lesions detected more than 1 year after curative CRT. All patients had previously undergone curative radiotherapy or CRT to the head and neck; the total prescribed dose to the previously irradiated area was 60–70 Gy. Induction chemotherapy consisted of TPF therapy (docetaxel + cisplatin + 5-FU), and curative CRT consisted of either CDDP monotherapy or FP therapy (5-FU + CDDP). All lesions were histologically confirmed as sHNSCC using biopsy specimens before ELPS, according to World Health Organization criteria [17].

The indications for ELPS in sHNSCC were limited to first-time lesions without muscle invasion, cervical lymph node metastasis, or distant metastasis, as assessed using computed tomography (CT) and positron-emission tomography (PET)-CT. Endoscopic evaluation was performed using white light endoscopy (GIF-H290Z; Olympus Co., Ltd., Tokyo, Japan) to assess tumor size and macroscopic type (0-Is, 0-Ip, 0-IIa, 0-IIb, or 0-IIc). In patients with multiple lesions, the primary lesion was defined based on subepithelial epithelial proliferation (SEP) and greater tumor thickness. Endoscopic findings were assessed by experienced endoscopists (SN and HY).

The feasibility and indications for ELPS were determined through multidisciplinary discussion involving an endoscopist and an otolaryngologist in all cases. Furthermore, the otolaryngologist discussed treatment options, including CRT and surgery, with a radiologist.

The indications for endoscopic resection (ER) of SCC were as follows: (1) histological confirmation of SCC, (2) absence of ulcerative findings suggestive of deep submucosal invasion, (3) no invasion of the bilateral pharyngeal or deep laryngeal spaces, and (4) no evidence of distant or lymph node metastasis on CT. The inclusion criteria for this study were histologically confirmed SCC confined to the epithelium or subepithelium in the resected specimen and no evidence of distant or lymph node metastasis on imaging. Because the laryngopharynx lacks a distinct muscularis mucosae and submucosal layer, the depth of invasion was assessed preoperatively by magnifying endoscopy with narrow-band imaging (ME-NBI), using the intrapapillary capillary loop (IPCL) pattern and the endoscopically estimated tumor thickness, according to the criteria of Katada et al. [18]. Lesions endoscopically judged as confined within the epithelium or subepithelium (without deep invasion) were considered eligible. In the two lesions with positive vertical margins, preoperative ME-NBI showed no findings of deep invasion, and these cases were therefore considered eligible.

This study was approved by the Institutional Review Board of Tokyo Medical University Hospital and was conducted in accordance with the principles of the 1964 Declaration of Helsinki and its later amendments. The Human Research Committee of the institution granted prior approval. A document describing the opt-out policy was implemented and publicly disclosed on the Tokyo Medical University Hospital website.

### ELPS procedure

ELPS was performed under general anesthesia, with the patient in the supine position. A head and neck surgeon inserted a rigid laryngoscope (Nagashima, Tokyo, Japan) to expand the pharyngeal space. Lesion margins were identified using magnifying endoscopy with narrow-band imaging (NBI) and iodine staining. A curved electrosurgical knife (KD-600; Olympus Co., Ltd., Tokyo, Japan) was used to mark a 5-mm margin around the unstained area.

To elevate the lesion, the surgeon injected saline mixed with indigo carmine and epinephrine into the subepithelial layer. While applying traction using curved grasping forceps, dissection and resection were performed with the electrosurgical knife. After resection, the mucosa was examined, and patients were extubated on the same day if no significant swelling was observed.

### Pathological evaluation

All resected specimens were sliced longitudinally into 2-mm sections, fixed in 10% formalin, and embedded in paraffin. Histopathological evaluation included tumor type, maximum diameter, lymphatic and vascular invasion, tumor thickness, epithelial thickness, and invasion depth.

Superficial pharyngeal cancer was defined as carcinoma confined to the epithelium or subepithelium without invasion of the muscularis propria. SEP was diagnosed when the tumor extended beyond the epithelial layer. Tumor diameter was measured microscopically at its maximum width. Squamous intraepithelial lesions were classified according to the WHO Classification of Head and Neck Tumors (4th edition). Dysplasia (low- and high-grade) was distinguished from squamous cell carcinoma in situ (Tis). Tis was defined as full-thickness cytological and architectural atypia of the epithelium without invasion beyond the basement membrane, whereas high-grade dysplasia was defined as severe atypia not involving the full epithelial thickness. Lesions diagnosed as dysplasia alone were excluded, and only lesions histologically confirmed as squamous cell carcinoma (Tis or invasive) were included. Lymphatic and vascular invasion were assessed using hematoxylin and eosin staining, with D2-40 immunostaining and elastic fiber staining used as needed. Tumor thickness was defined as the distance from the mucosal surface to the deepest point of invasion, measured at the site of maximum depth. All specimens were centrally reviewed by certified pathologists.

### Post-treatment follow-up surveillance

Surveillance included white-light endoscopy and NBI in all cases. Lymph node metastasis was assessed every 2–3 months via ultrasound and CT of the neck and every 6 months via chest CT.

### Definition of cancer recurrence

Patients who underwent preoperative treatment for superficial lesions before endoscopic treatment were followed up using CT every 3 months to monitor for metastasis. During follow-up, the presence of residual tumor tissue or new malignant lesions (metachronous or recurrent) was assessed. We defined local recurrences as lesions occurring near the primary site, such as those at the margin of the post-resection scar.

### Statistical analysis

Quantitative data were compared using the Mann–Whitney U test. Categorical data were analyzed using Fisher’s exact and χ^2^ tests. Overall survival (OS) was defined as the time from ER until death or the last follow-up. Survival time was measured from the date of ER to the date of death or the latest survival confirmation before 2024. For analysis of metachronous sHNSCC, follow-up was censored at the last endoscopy examination before 2024. The time to occurrence of metachronous sHNSCC was defined as the interval from the date of ER to the date of detection.

The cumulative incidence of metastatic sHNSCC was estimated for the first metastatic event, and survival curves were generated using the Kaplan–Meier method. Statistical analyses were performed using IBM SPSS Statistics for Windows, version 29.0 (IBM Japan, Tokyo, Japan), with statistical significance set at *p* <0.05. Postoperative adverse events were graded according to the Clavien–Dindo classification [19].

## Results

### Patient characteristics

This study included 17 patients (15 males and 2 females). The median age was 71.2 years (range, 63.2–78.2), and six patients (35.3%) were >75 years. Regarding treatment indications, 10 and seven patients underwent salvage ELPS for sHNSCC and metachronous ELPS, respectively. The median observation period was 47.5 months (range, 14.1–99.9 months).

Assessment of nutritional and clinical status showed that three patients (17.6%) had a body mass index (BMI) <18.5 kg/m^2^, ten (58.8%) had a prognostic nutritional index (PNI) <40, and three (17.6%) had serum albumin levels <3.5 g/dL.

Regarding alcohol consumption, six patients (35.3%) were heavy drinkers, five (29.4%) were moderate drinkers, five (29.4%) were light drinkers, and one (5.9%) reported never or rarely consuming alcohol. Regarding smoking history, three patients (17.6%) were non-smokers, ten (58.8%) had a smoking index <30 pack-years, and four (23.5%) had ≥30 pack-years. Double primary carcinomas were observed in six patients (35.3%), including ESCC in three patients (17.6%) and gastric cancer in one patient (5.9%). Among the three ESCC cases, two were treated with endoscopic submucosal dissection before ELPS and one underwent surgical (esophagectomy) resection after ELPS. Preoperative lesion characteristics showed that most lesions were located in the pharynx 14 (82.4%): 8 in pyriform sinus (47.1), 4 in posterior wall (23.5%), and 2 in the postcricoid area (11.8%). The larynx (supraglottis) had one (5.9%) and the oropharynx had two (11.8%): a superior one (5.9%) and an anterior one (5.9%). The JES classification of vascular atypia showed types B1, B2, B3, and unknown in seven (41.2%), six (35.3%), three (17.6%), and one (5.9%) of the patients, respectively. Clinical staging before CRT revealed stage I/II/Ⅲ/ Ⅳ/ unknown in one (5.9%), eight (47.1%), two (11.8%), one (5.9%) and four (23.5%) patients, respectively. Clinical staging at the time of salvage ELPS revealed Tis/T1/T2 disease in one (5.9%), 10 (58.8%), and six (35.3%) patients, respectively (Table 1).

**Table 1:** Characteristics of patients with superficial squamous cell head and neck cancer

Patients	n=17
Age (years), median (range)	71.2 (63.2–78.2)
Age >75 (years), n (%)	6 (35.3)
Sex, male/female	15/2
Salvage/metachronous	10/7
Observation periods, median (range), months	47.5 (14.1–99.9)
BMI (< 18.5 kg/m^2^)	3 (17.6)
PNI (< 40), n (%)	10 (58.8)
NLR (> 3.0), n (%)	9 (52.9)
GPS score (0/1), (%)	82.4/17.6
Albumin (< 3.5 g/dL)	3 (17.6)
*Alcohol consumption*	
Heavy, n (%)	6 (35.3)
Moderate, n (%)	5 (29.4)
Light, n (%)	5 (29.4)
Never/Rare, n (%)	1 (5.9)
*Smoking*	
Non-smoker	3 (17.6)
<30 pack-years	10 (58.8)
≥30 pack-years	4 (23.5)
Duplicated carcinoma	6 (35.3)
ESCC	3 (17.6)
GC	1 (5.9)
Preoperative characteristics of lesions	
*Location*	
Pharynx, n (%) (pyriform sinus/posterior wall/postcricoid area), n	14 (82.4) (8/4/2)
Larynx, n (%) (glottis/supraglottis), n	1 (5.9) (0/1)
Oropharynx, n (%) (superior/lateral/posterior/anterior wall), n	2 (11.8) (1/0/0/1)
JES-SCC Type-B1/B2/B3/unknown, n	7/6/3/1
Clinical stage before CRT, n (I/II/Ⅲ/Ⅳ/unknown)	1/8/2/3/3
Clinical stage at the time of salvage ELPS, n (Tis/T1/T2)	1/10/6

^a^Never, <1 U/week; light, 1–9 U/week; moderate, >9–18 U/week; heavy, >18 U/week, where U = 22 g of ethanol

BMI, body mass index; PNI, prognostic nutritional index; ELPS, endoscopic laryngopharyngeal surgery; NLR, neutrophil-to-lymphocyte ratio; GPS, Glasgow Prognostic Score; ESCC, esophageal squamous cell carcinoma; GC, gastric cancer; CRT, chemoradiotherapy

**Fig. 1:**
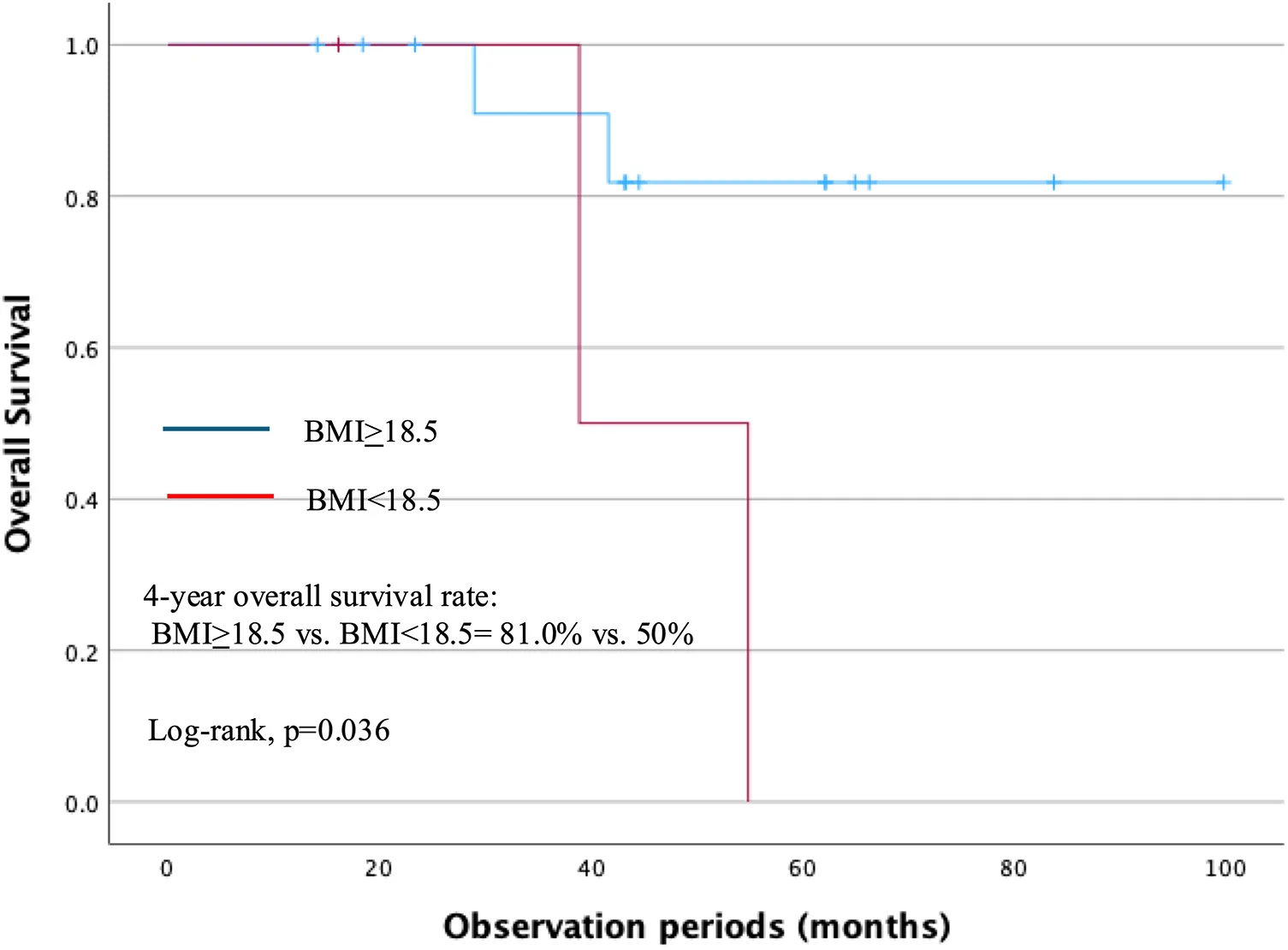
Kaplan–Meier curve of overall survival stratified by BMI. Patients with BMI ≥18.5 kg/m^2^ demonstrated significantly better overall survival than those with BMI <18.5 kg/m^2^ (4-year OS: 81.8% vs. 50%; log-rank *p*=0.036). BMI, body mass index

Kaplan–Meier analysis demonstrated a significant association between nutritional status and OS. Patients with BMI ≥18.5 kg/m^2^ had a markedly better prognosis than those with BMI<18.5 kg/ m^2^. The OS differes significantly between patients with BMI ≥18.5 kg/m^2^ and those with BMI<18.5 kg/ m^2^ (4-year OS rates: 81.8%; vs. 50%, *p*=0.036) (Fig. 1).

### Lesion characteristics and treatment outcomes

The median lesion size was 18.1 mm (range, 8.4–27.8 mm). The en bloc resection rate was 100%. The median treatment time was 59.3 min (range, 33.5–85.1 min), and 10 procedures (58.8%) were completed within 60 min.

Regarding macroscopic appearance, the lesions were classified as type 0–I in four patients (23.5%), type 0-IIa in seven (41.2%), type 0-IIb in three (17.6%), and type 0-IIc in three (17.6%).

Pathological assessment revealed carcinoma in situ (CIS) in six lesions (35.3%) and SEP in 11 lesions (64.7%). Tumor thickness >1000 μm was observed in six lesions (35.3%).

Negative horizontal margins were achieved in 12 lesions (70.6%), whereas five lesions (29.4%) had positive horizontal margins. Negative vertical margins were observed in 15 lesions (88.2%) and positive vertical margins in two lesions (11.8%). The two lesions with positive vertical margins received no additional treatment immediately after ELPS and were managed with close surveillance; one of these subsequently developed a metachronous recurrence that was treated by repeat ELPS. Lymphatic invasion was identified in four lesions (23.5%), and vascular invasion in five lesions (29.4%).

AEs occurred in eight patients (47.1%), including laryngeal edema (n=3, 17.6%), pneumonia (n=1, 5.9%), stenosis (n=1, 5.9%), hemorrhage (n=1, 5.9%), and recurrent laryngeal nerve palsy (n=2, 11.8%) (Table 2). All 10 salvage cases were local recurrences after a clinical complete response. The median postoperative hospital stay was 10.4 days, and the median time to resume oral intake was 2.8 days. Two patients required temporary nasogastric tube feeding (until postoperative day 7 and day 19, respectively) before resuming oral intake; no patient required gastrostomy or permanent feeding-tube dependence. Regarding laryngeal function, one patient who developed postoperative aspiration pneumonia required a temporary tracheostomy, which was closed after recovery; this patient resumed oral intake without the need for swallowing rehabilitation. No patient had vocal fold paralysis, and swallowing function was preserved in all cases. Adverse events were graded by the Clavien–Dindo classification [19]; no Grade IV/V events occurred.

**Table 2:** Treatment outcomes of ELPS

Patients	n=17
Size (mm), median (range)	18.1 (8.4–27.8)
En bloc resection rate, %	100
Treatment time (minutes), median (range)	59.3 (33.5–85.1)
Treatment time ≤60 min	10 (58.8)
*Macroscopic type*	
0-I, n (%)	4 (23.5)
0-IIa, n (%)	7 (41.2)
0-IIb, n (%)	3 (17.6)
0-IIc, n (%)	3 (17.6)
*Pathological diagnosis depth, n (%)*	
CIS, n (%)	6 (35.3)
SEP, n (%)	11 (64.7)
Tumor thickness (≥ 1000 μm), n (%)	6 (35.3)
*Horizontal margin*	
Negative, n (%)	12 (70.6)
Positive, n (%)	5 (29.4)
*Vertical margin*	
Negative, n (%)	15 (88.2)
Positive, n (%)	2 (11.8)
*Lymphatic invasion*	
Negative, n (%)	13 (76.5)
Positive, n (%)	4 (23.5)
*Vascular invasion*	
Negative, n (%)	12 (70.6)
Positive, n (%)	5 (29.4)
Adverse events, n (%)	8 (47.1)
Clavien–Dindo classification, n I/II/Ⅲa/Ⅲb/Ⅳ/V	5/0/3/0/0
Laryngeal edema, n (%)	3 (17.6)
Pneumonia, n (%)	1 (5.9)
Stenosis, n (%)	1 (5.9)
Hemorrhage, n (%)	1 (5.9)
Recurrent nerve palsy, n (%)	2 (11.8)

ELPS, endoscopic laryngopharyngeal surgery; CIS, carcinoma in situ; SEP, subepithelial proliferation

### Comparison between salvage and metachronous ELPS

Patient characteristics did not differ significantly between the salvage ELPS for sHNSCC (n=10) and metachronous ELPS (n=7) groups. We observed no significant differences between the two groups in baseline characteristics, including age (median 73.6 vs. 67.9 years, *p*=0.574), proportion of patients aged >75 years (30.8% vs. 50%, *p*=0.458), sex distribution (*p*=0.154), BMI <18.5 kg/m^2^ (*p*=1.000), observation period (median 46.5 vs. 50.5 months, *p*=0.386), alcohol consumption patterns (*p*=0.151), smoking status (*p*=0.537), and frequency of duplicate carcinomas, including ESCC (*p*=1.000).

Regarding endoscopic treatment outcomes, tumor size (median 20.9 vs. 13.1 mm), tumor location, and en bloc resection rates (100% in both groups) were similar. The median treatment time tended to be longer in the salvage group (69.4 vs. 44.9 min), although the difference was not statistically significant (*p*=0.053). The proportion of procedures completed within 60 min was also comparable, and pathological findings, including invasion depth, tumor thickness, margin status, lymphatic invasion, and vascular invasion, did not differ significantly between the groups.

In contrast, AEs occurred significantly more frequently in the salvage ELPS for sHNSCC group than in the metachronous ELPS group (70% vs. 14.3%, *p*=0.024). Individual AEs, including laryngeal edema, pneumonia, stenosis, hemorrhage, and recurrent nerve palsy, did not show significant differences (Table 3). According to the Clavien–Dindo classification, laryngeal edema, stenosis, and recurrent laryngeal nerve palsy were classified as Class I, while aspiration pneumonia and bleeding were classified as Class IIIa.

**Table 3:** Comparison of clinical characteristics, treatment outcomes, and adverse events between salvage and metachronous ELPS

	Salvage ELPS	Metachronous ELPS	*p*-value
	n=10	n=7	
*Patients*			
Age (years), median (range)	73.6 (67.0–80.3)	67.9 (58.7–77.0)	0.574
Age (years) >75	4 (30.8)	2 (50)	0.458
Sex, male/female	10/0	5/2	0.154
BMI <18.5 kg/m^2^	2 (20)	1 (14.3)	1.000
Observation periods, median (range), months	46.5 (19.5–73.5)	50.5 (38.7–62.3)	0.386
*Alcohol consumption*			
Heavy, n (%)	5 (50)	1 (14.3)	0.151
Moderate, n (%)	3 (30)	2 (28.6)	
Light, n(%)	1 (10)	4 (57.1)	
Never/rarely, n (%)	1 (10)	0	
*Smoking*			
Non-smoker	1 (10)	2 (28.6)	0.537
<30 pack-years	6 (60)	4 (57.1)	
≥30 pack-years	3 (30)	1 (14.3)	
Duplicated carcinoma	4 (40)	2 (28.6)	1.000
ESCC	2 (20)	1(14.3)	1.000
*Endoscopic treatment*			
Size (mm), median (range)	20.9 (8.7–33.1)	13.1 (8.3–18.0)	0.324
Postoperative stay (range), days	12.4 (7–38)	7.6 (5–12)	0.223
The time to resume oral intake (range), days	3.1 (1–19)	2.3 (1–6)	0.476
*Location*			
Pharynx, n (%)	9 (90)	5 (71.4)	0.435
Larynx, n (%)	0	1 (14.3)	
Oropharynx, n (%)	1 (10)	1 (14.3)	
En bloc resection rate, %	10 (100)	7 (100)	–
Treatment time (minutes), median (range)	69.4 (44.3–94.5)	44.9 (24.6–65.1)	0.053
Treatment time <60 min	5 (50)	5 (71.4)	0.622
*Pathological diagnosis depth, n (%)*			
CIS, n (%)	2 (20)	4 (57.1)	0.162
SEP, n (%)	8 (80)	3 (42.9)	
Tumor thickness (≥ 1000 μm), n (%)	5 (50)	2 (28.6)	0.622
*Horizontal margin*			
Negative	6 (60)	6 (85.7)	0.394
Positive	2 (20)	0	
Unclear	2 (20)	1 (14.3)	
*Vertical margin*			
Negative	10 (100)	5 (71.4)	0.154
Positive	0	2 (28.6)	
*Lymphatic invasion*			
Negative	7 (70)	7 (100)	0.228
Positive	3 (30)	0	
*Vascular invasion*			
Negative	6 (60)	6 (85.7)	0.338
Positive	4 (40)	1 (14.3)	
Adverse events, n (%) Clavien–Dindo classification, n (%) (I/II/Ⅲa/Ⅲb/Ⅳ/Ⅴ)	7 (70)	1 (14.3)	0.024
Laryngeal edema	2 (20) (2/0/0/0/0/0)	2 (28.6) (2/0/0/0/0/0)	0.304
Pneumonia	1 (10) (0/0/1/0/0/0)	0	0.304
Stenosis	1 (10) (1/0/0/0/0/0)	0	0.304
Hemorrhage	1 (10) (0/0/1/0/0/0)	0	0.304
Recurrent nerve palsy	2 (20) (2/0/0/0/0/0)	0	0.304

^a^Never, <1 U/week; light, 1–9 U/week; moderate, >9–18 U/week; heavy, >18 U/week, where U = 22 g ethanol

ELPS, endoscopic laryngopharyngeal surgery; ESCC, esophageal squamous cell carcinoma; CIS, carcinoma in situ; SEP, subepithelial proliferation

**Fig. 2:**
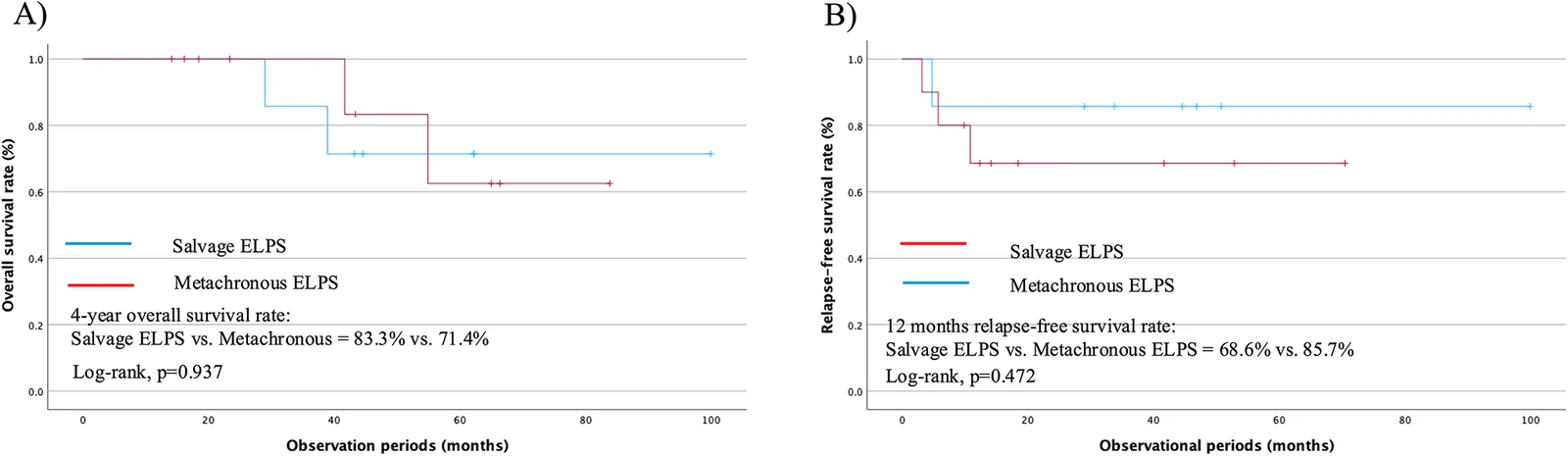
Overall and relapse-free survival stratified by treatment group. **a** OS stratified by group (salvage ELPS for sHNSCC vs. metachronous ELPS). OS does not differ significantly between the two groups (4-year OS: 83.3% vs. 71.4%, respectively; log-rank *p*=0.937). Median survival was not reached in either group during the observation period. **b** RFS stratified by treatment group (salvage ELPS for sHNSCC vs. metachronous ELPS) showing no significant difference (68.6% vs. 85.7%, log-rank *p*=0.472). OS, overall survival; ELPS, endoscopic laryngopharyngeal surgery; RFS, relapse-free survival

Regarding survival outcomes, the OS rate did not differ significantly between the salvage and metachronous ELPS groups (4-year OS: 83.3% vs. 71.4%, *p*=0.937). The median survival was not reached in either group during the observation period (Fig. 2a). Similarly, relapse-free survival (RFS) did not differ significantly between the salvage and metachronous ELPS groups (12-month RFS: 68.6% vs. 85.7%, *p*=0.472), with no statistically significant difference in early disease control between the two groups (Fig. 2b).

### Adverse events

Eight patients (47.1%) experienced AEs following ELPS. Salvage ELPS for sHNSCC accounted for seven of the eight patients who developed AEs. On univariate analysis, salvage status (*p*=0.024) and low BMI (*p*=0.043) were significantly associated with AE occurrence (Table 4). Other clinical factors, including age >75 years, sex, PNI <40, NLR >3.0, Glasgow Prognostic Score (GPS) (0/1), albumin <3.5 g/dL, heavy alcohol consumption, and heavy smoking, were not significantly associated with AEs. Tumor-related and procedural factors, including location, tumor size ≥20 mm, treatment time ≥60 min, synchronous lesions, macroscopic type, depth of invasion, and tumor thickness ≥1000 μm, were also not significantly associated with AEs (Table 4).

**Table 4:** Factors associated with adverse events following ELPS

Patients	n=8	*p*-value
*Patient characteristics*		
Age (years) >75	2 (25)	0.402
Sex, male/female	7 (87.5)/1 (12.5)	0.929
Salvage/metachronous	7 (87.5)/1 (12.5)	0.024
BMI<18.5 kg/m^2^, n (%)	3 (37.5)	0.043
PNI (< 40), n (%)	5 (62.5)	0.772
NLR (> 3.0), n (%)	4 (50)	0.819
GPS score (0/1), (%)	0	0.072
Albumin (< 3.5 g/dL)	0	0.072
*Alcohol consumption*		
Heavy, n (%)	4 (50)	0.232
*Smoking*		
≥30 pack-years	3 (37.5)	0.200
*Location*		
Pharynx, n (%)	8 (100)	0.198
Larynx, n (%)	0	
Oropharynx, n (%)	0	
*Treatment outcome*		
Size(mm) ≥20	3 (37.5)	0.490
Treatment time ≤60 min	5 (62.5)	0.772
Synchronous lesion	1 (12.5)	0,274
*Macroscopic type*		
0-I, n (%)	2 (25)	0.861
0-IIa, n (%)	3 (37.5)	
0-IIb, n (%)	1 (12.5)	
0-IIc, n (%)	2 (25)	
*Pathological diagnosis depth, n (%)*		
CIS/SEP, n (%)	2 (25)/6 (75)	0.402
Tumor thickness ≥1000 μm	4 (50)	0.486

ELPS, endoscopic laryngopharyngeal surgery; BMI, body mass index; PNI, prognostic nutritional index; NLR, neutrophil-to-lymphocyte ratio; GPS, Glasgow Prognostic Score; CIS, carcinoma in situ; SEP, subepithelial proliferation

## Discussion

Salvage ELPS for sHNSCC is a technically demanding procedure associated with a higher incidence of AEs due to prior treatment-related tissue changes; however, it achieves oncologic outcomes comparable to those of metachronous ELPS and represents a feasible organ-preserving strategy, particularly when appropriate patient selection and nutritional status are considered.

### Salvage ELPS for sHNSCC

Our results demonstrated that salvage ELPS for sHNSCC was associated with a significantly higher incidence of AEs compared with metachronous ELPS, whereas oncogenic outcomes, including OS, were comparable between the two groups. Thus, although salvage ELPS for sHNSCC is technically more demanding and carries an increased procedural risk, it remains a feasible and effective organ-preserving treatment option.

Although salvage ELPS for sHNSCC is associated with an increased risk of perioperative AEs attributable to tissue changes following CRT, it achieves long-term survival outcomes comparable to those of metachronous ELPS. The 100% en bloc resection rate in both groups indicated equivalent technical feasibility. The 4-year OS rates did not differ significantly between the salvage and metachronous groups. Although the incidence of AEs was significantly higher in the salvage group, this increase in perioperative risk did not translate into inferior survival outcomes. Taken together, these findings support salvage ELPS for sHNSCC as a feasible organ-preserving therapeutic strategy for residual or recurrent sHNSCC, offering a favorable balance between increased procedural risk and long-term prognosis. Functional organ preservation was supported by the postoperative course: no patient had vocal fold paralysis, swallowing function was preserved in all cases, and only one patient required a temporary tracheostomy (for aspiration pneumonia) that was subsequently closed, with no permanent tracheostomy or gastrostomy dependence. These functional outcomes are consistent with previous reports of ELPS for superficial pharyngeal cancer, in which voice and swallowing functions were preserved in the majority of patients [20]. When graded with the Clavien–Dindo classification, the majority of adverse events were low-grade (grade I–II) and managed conservatively; although a subset required endoscopic or surgical intervention (grade IIIa/IIIb), no grade IV or V event occurred. This severity profile indicates that the higher adverse-event frequency in the salvage setting was driven mainly by manageable complications rather than life-threatening events, further supporting the feasibility of salvage ELPS for sHNSCC.

### Radiation effects and procedural risk in salvage ELPS

The higher complication rate observed in the salvage group was likely attributable to treatment-related tissue changes. Patients who undergo salvage ELPS for sHNSCC often have a history of CRT, which can induce fibrosis, microvascular damage, and chronic inflammation. These alterations reduce tissue elasticity, obscure anatomical planes, and impair wound healing, thereby increasing the risk of complications such as laryngeal edema, bleeding, and nerve injury [21, 22]. This interpretation is supported by the observed trend toward longer procedure times and postoperative hospital stay in the salvage group, reflecting the increased technical complexity of this technique. Additionally, the tendency toward longer procedure times in the salvage group may reflect the obscuration of the anatomical layers caused by post-radiation changes. These findings are consistent with previous reports describing radiation-induced tissue alterations such as fibrosis, edema, and xerostomia [16]. Taken together, the increased risk observed in the salvage setting appears predictable and should be carefully assessed and anticipated during preoperative planning.

### Comparable survival despite increased risk in salvage ELPS

Despite the increased perioperative risk observed in the salvage group, 4-year OS and median survival did not differ significantly between the two groups, indicating that salvage ELPS for sHNSCC provides adequate local control, even in previously irradiated fields. In particular, a numerically higher 4-year OS rate (83.3% vs. 71.4%) was observed in the salvage group; however, this should not be interpreted as a true survival advantage against metachronous disease. Rather, given the small size of the subgroup and the median follow-up period of 47.5 months, reporting the 4-year OS rather than the 5-year OS was intended to avoid the less reliable terminal portion of the survival curve, where only a very small number of at-risk patients remain. Although the 12-month RFS rate was lower in the salvage group, suggesting a tendency toward earlier recurrence, this did not appear to substantially impact long-term survival outcomes. Thus, even in cases of early recurrence, appropriate post-salvage surveillance and additional interventions may help maintain a favorable long-term prognosis.

### Nutritional status determines outcomes in salvage ELPS for sHNSCC

Low BMI (< 18.5 kg/m^2^) was an independent predictor of both AE occurrence and 4-year OS, positioning nutritional assessment as the central determinant of patient selection and perioperative management in salvage ELPS for sHNSCC. CRT and RT are important treatment strategies for HNSCC from the perspective of functional preservation. However, approximately 40–60% of patients experience locoregional recurrence [5]. In patients with resectable recurrent disease, salvage surgery is a potential option but is often associated with considerable morbidity and suboptimal functional outcomes [23]. In contrast, re-irradiation is limited by cumulative toxicity, deterioration in quality of life, and dose constraints, thereby restricting its clinical applicability [24]. Therefore, the role of organ-preserving approaches such as ELPS in salvage settings warrants careful consideration. Recent advances in endoscopic imaging, particularly NBI, have enabled earlier detection of sHNSCC [25]. This has facilitated the implementation of structured surveillance strategies following initial treatment, allowing detection at a stage amenable to organ-preserving interventions such as ELPS. Such an approach may not only reduce the treatment-related burden but also contribute to improved long-term outcomes.

Importantly, our study demonstrated that a low BMI was significantly associated with both AEs and OS. Patients with BMI <18.5 kg/m^2^ experienced a higher incidence of complications and a markedly worse prognosis (*p*=0.036). Our results suggest that salvage ELPS is a valuable treatment option from the perspectives of safety, recurrence control, and survival, particularly when combined with appropriate patient selection and nutritional assessment. The integration of organ-preserving salvage strategies with early detection and optimized supportive care may further improve outcomes in this patient population. Taken together, salvage ELPS for sHNSCC preceded by rigorous patient selection, particularly nutritional assessment, and supported by optimized perioperative care, can serve as a reliable organ-preserving strategy for the management of post-CRT sHNSCC.

This study has some limitations. First, the retrospective single-center study design and small sample size limited the statistical power. Second, selection bias may have influenced the results, as patients selected for ELPS were likely to have a relatively favorable performance status and resectable superficial disease. Finally, although BMI was identified as a significant factor associated with both AEs and OS, more comprehensive assessments of nutritional and frailty statuses, such as sarcopenia or functional measures, were not included and warrant further investigation. Moreover, only three patients had a BMI <18.5 kg/m^2^, and this small number limits the strength of any causal inference. The association between low BMI and outcomes should therefore be interpreted as an observed, hypothesis-generating finding rather than evidence of direct causality, and requires confirmation in larger cohorts.

In conclusion, salvage ELPS for sHNSCC was associated with a higher risk of AEs but achieved long-term survival outcomes comparable to those of metachronous ELPS. Nutritional status, particularly a low BMI, appeared to be an important prognostic factor that may influence treatment safety. Since laryngectomy was avoided in all cases, these findings support the feasibility of ELPS as an organ-preserving approach while highlighting the need for careful patient selection and perioperative management, especially in the salvage setting.

## Data Availability

The datasets generated during and/or analyzed in this paper are available from the corresponding author on reasonable request.
